# Application of Unilateral Pectoralis Major Muscle Flap in the Treatment of Sternal Wound Dehiscence

**DOI:** 10.21470/1678-9741-2017-0038

**Published:** 2017

**Authors:** Grazielle de Souza Horácio, Pedro Soler Coltro, Antonio Albacete Neto, Juliano Baron Almeida, Vinícius Zolezi da Silva, Ivan de Rezende Almeida, Alfredo José Rodrigues, Jayme Adriano Farina Junior

**Affiliations:** 1 Division of Plastic Surgery, Faculdade de Medicina de Ribeirão Preto da Universidade de São Paulo (FMRP-USP), Ribeirão Preto, SP, Brazil.; 2 Division of Thoracic and Cardiovascular Surgery, Faculdade de Medicina de Ribeirão Preto da Universidade de São Paulo (FMRP-USP), Ribeirão Preto, SP, Brazil.

**Keywords:** Surgical Flaps, Surgical Wound Dehiscence, Sternum, Mediastinal Thoracic Wall

## Abstract

**Objective:**

This study aims to report the use of the unilateral pectoralis major muscle flap for the treatment of the sternal wound dehiscence.

**Methods:**

A retrospective study including patients who underwent unilateral pectoralis major muscle flap was performed for the treatment of sternotomy dehiscence due to coronary artery bypass, valve replacement, congenital heart disease correction and mediastinitis, between 1997 and 2016. Data from the epidemiological profile of patients, length of hospital stay, postoperative complications and mortality rate were obtained.

**Results:**

During this period, 11 patients had their dehiscence of sternotomy treated by unilateral pectoralis major muscle flap. The patients had a mean age of 54.7 years, the mean hospital stay after flap reconstruction was 17.9 days (from 7 to 52 days). In two patients, it was necessary to harvest a flap from the rectus abdominis fascia, in association with the pectoralis major muscle flap, to facilitate the closure of the distal wound. In the postoperative period, seroma discharge from the surgical wound was observed in six patients, five reported intense pain (temporary), three had partial cutaneous dehiscence, and two presented granuloma of the incision.

**Conclusion:**

The complex wound from sternotomy dehiscences presents itself as a challenge to surgical teams. Treatment should include debridement of necrotic tissue and preferably coverage with well-vascularized tissue. We propose that the unilateral pectoralis major muscle flap is an interesting and low morbidity option for the reconstruction of sternal wound dehiscences, with proper sternum stability and satisfactory functional and aesthetic outcomes.

## INTRODUCTION

In the 1950s, Shumacker and Lurie^[1]^ introduced the median sternotomy as an access route for cardiac surgery, including coronary artery bypass procedures^[2]^. Although the incidence of infection in the median sternotomy is relatively low (1-10%), when associated with mediastinitis, it courses with high morbidity and mortality rate ranging from 14 to 47%^[3,4]^. Besides, sternal wound dehiscence could often lead to major defects of the anterior chest wall, and it could expose the heart, vessels or any vascular prostheses and coronary grafts^[3]^. Also, the resulting chest wall instability impairs respiratory function. Therefore, such patients commonly require prolonged mechanical ventilation, and experience a difficulty of the tissue healing^[5]^.

In great chest wounds, especially those resulting from infection, the transposition of vascularized tissues to the affected area greatly contributes to the stabilization of the chest wall, and therefore to the ventilation dynamics, as well as to overcome the infection and to accelerate the healing^[4]^. Besides, flap transposition also contributes to aesthetics outcomes.

Among the various methods used for the treatment of the sternal wound dehiscence, medical literature highlights the utilization of the bilateral pectoralis major muscle flap^[2,3,6,7]^. Although it is possible to use only one of the pectoralis major muscles, there are scarce sources of literature about the application of the unilateral pectoralis major muscle flap^[8-11]^, based on the thoracoacromial artery.

The purpose of this article is to report the use of the unilateral pectoralis major muscle flap for the treatment of the sternal wound dehiscence.

## METHODS

A retrospective study was performed with 11 patients undergoing a late reconstruction of sternal wound dehiscence using the unilateral pectoralis major muscle flap. These surgeries were performed at the Division of Plastic Surgery, Ribeirão Preto Medical School, University of São Paulo, Brazil, between 1997 and 2016. This study was approved by our Institutional Review Board.

We obtained data from medical charts: epidemiological profile of patients (age, sex, and comorbidities), length of hospital stay, drainage time, postoperative complications, mortality rate, and results of radiologic exams. Sternal stability was assessed by palpation on physical examination.

The inclusion criteria were: patients who underwent a late reconstruction of sternal wound dehiscence using the unilateral pectoralis major muscle flap, due to open-heart surgeries or secondary to infection. Patients in which the reconstruction was performed with other types of flaps or after ablation of sternal tumors were excluded.

### Operative Technique

All surgeries were performed by the same surgeon with dissection of the left pectoralis major muscle. The flaps were dissected using electrocautery and a blunt divulsion. They were deinserted from the sternal and costal region, remaining only adhered in the middle third of the clavicle, where the vascular pedicle of the thoracoacromial artery was located. For enhancing the arc of rotation, the muscle origin was sectioned from the humerus under direct vision with the aid of a small incision of about 4 cm at the deltopeitoral fold. After wide release, the muscle was mobilized and sutured with an unabsorbable suture to the sternal wound margins, with its medial border inserted into the sternal cleft to avoid dead space and fluid collection. After total obliteration of the sternal cleft, a suction drain was placed in the dissection area and cutaneous flaps were harvested bilaterally and advanced to the medial direction to close the wound.

## RESULTS

**Fig. 1:**
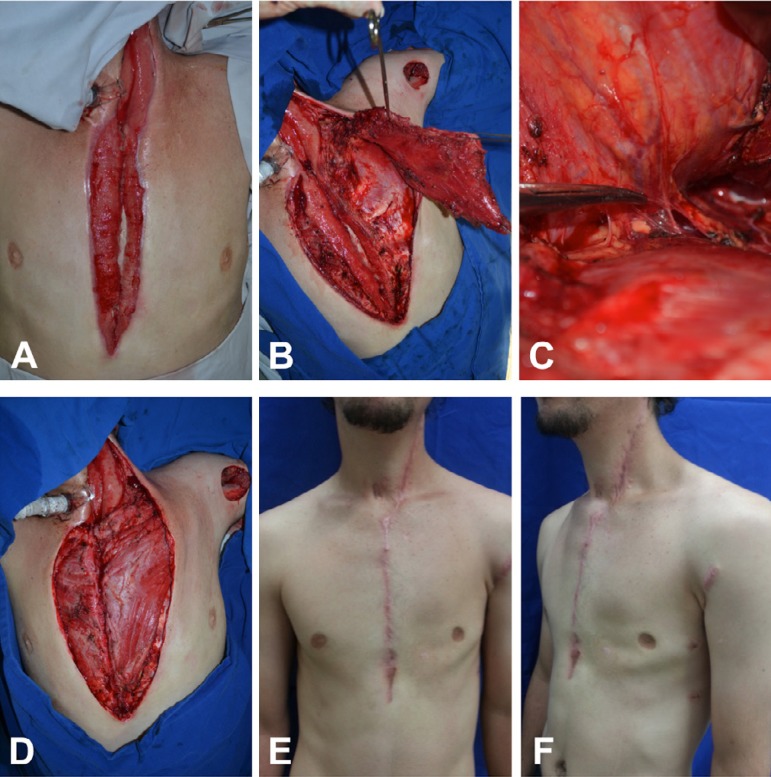
A 21-year-old man with sternal wound dehiscence due to mediastinitis after Ludwig's syndrome. (A) Cervical-sternal wound dehiscence. (B) Transposition of the left pectoralis major muscle flap after the section of its origin at the humerus. (C) Left thoraco-acromial pedicle. (D) Flap positioned and fixed, covering the entire extent of the sternal cleft. (E and F) Four months after surgery.

**Fig. 2:**
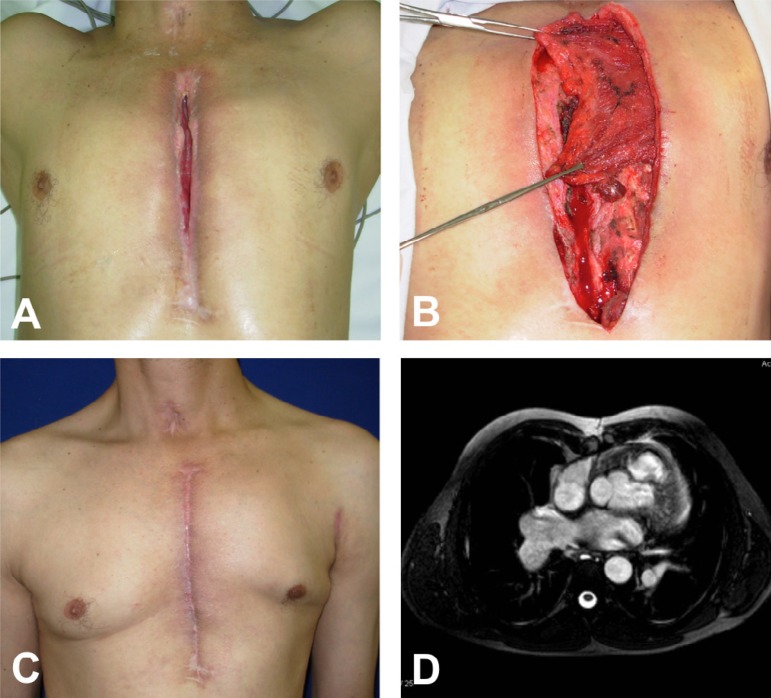
A 40-year-old man with sternal wound dehiscence due to correction of tetralogy of Fallot. (A) Sternal wound dehiscence. (B) Transposition of the left pectoralis major muscle flap after the section of its origin at the humerus. (C) Two months after surgery. (D) Computed tomography eight years surgery.

**Fig. 3:**
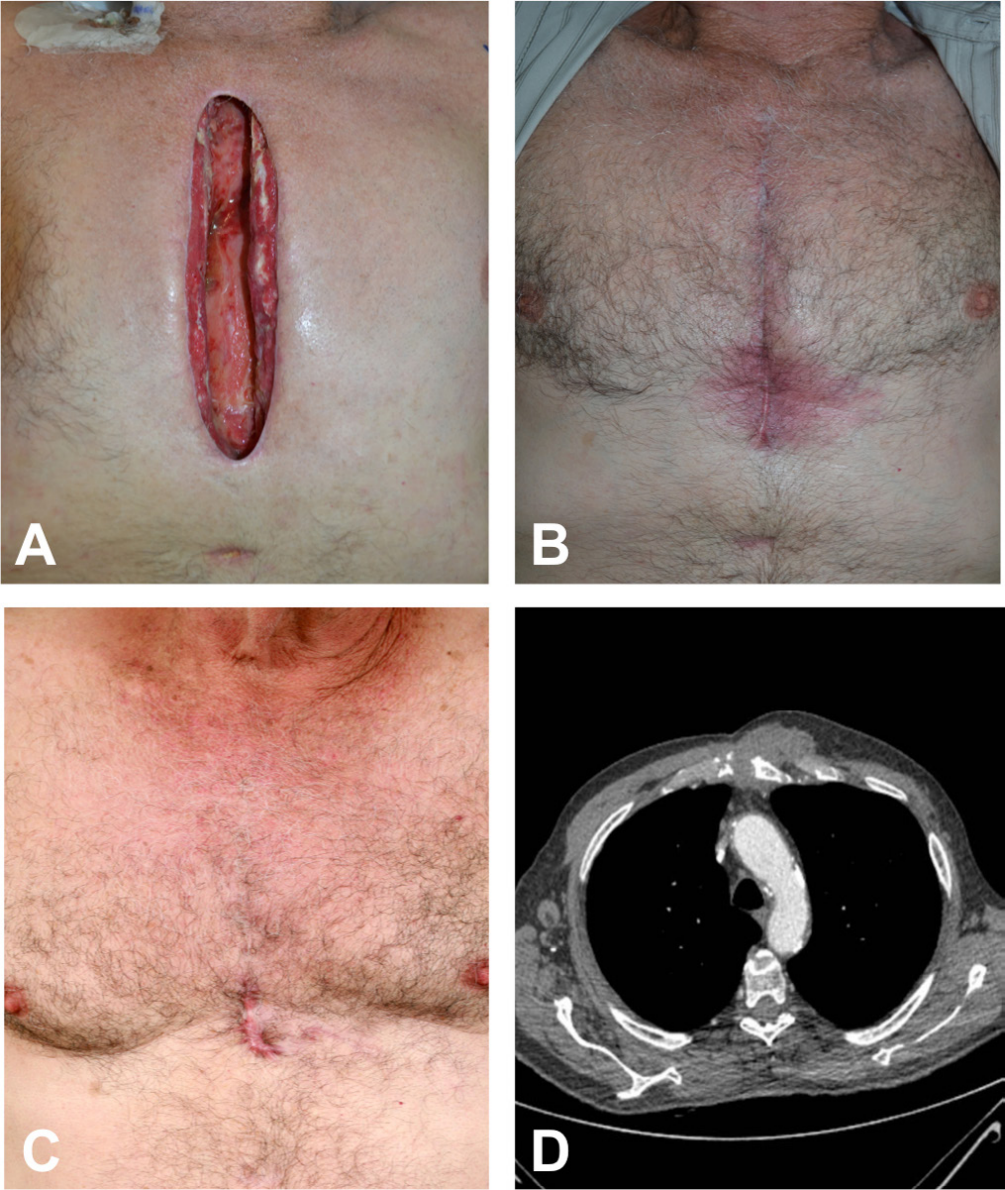
A 67-year-old man with sternal wound dehiscence due to mediastinitis after valve replacement surgery underwent reconstruction using the left pectoralis major muscle flap. (A) Sternal wound dehiscence. (B) Hyperemia of the lower scar due to subcutaneous granuloma three months surgery. (C) Three-month postoperative period after correction of the granuloma using local rhomboid flap. (D) Computed tomography six months after surgery.

Among the 11 patients operated, seven were due to sternotomy dehiscence after myocardial revascularization, one after valvuloplasty, one after valve replacement, one after correction of congenital heart disease (Tetralogy of Fallot) and one resulting from mediastinitis after Ludwig's syndrome (Figures 1 to 3).

The patients had a mean age of 54.7±13.6 years (from 21 to 67 years), eight were male and three female (Table 1). The mean hospital stay after flap reconstruction was 17.9±15.6 days (from 7 to 52 days) and the mean time of suction drain was 8.3±3.2 days (from 5 to 14 days). Patients had the following comorbidities: hypertension (n=9), diabetes (n=5), chronic obstructive pulmonary disease (n=3) and obesity (n=4). The mean follow-up time was 14.8 months (from 6 to 19 months).

**Table 1:** Data from the patients who underwent sternal wound closure using unilateral pectoralis major muscle flap.

Patient's age/gender	Previous surgery	Reconstruction/flap	Drainage time(days)	Postoperative complications	Length of hospital stay (days)
56/F	Valvuloplasty	Left pectoralis major muscle flap	5	__	37
62/M	Myocardial revascularization	Left pectoralis major muscle flap	5	Seroma, pain, wound infection	8
56/F	Myocardial revascularization	Left pectoralis major muscle flap	7	Partial dehiscence	52
66/M	Myocardial revascularization	Left pectoralis major muscle flap	7	__	32
40/M	Correction of tetralogy of Fallot	Left pectoralis major muscle flap	6	__	3
53/F	Myocardial revascularization	Left pectoralis major muscle flap + Abdominal rectus fascia flap	8	Pain	11
55/M	Myocardial revascularization	Left pectoralis major muscle flap + Abdominal rectus fascia flap	10	Seroma, pain	11
60/M	Myocardial revascularization	Left pectoralis major muscle flap	14	Seroma, pain, partial dehiscence, granuloma	8
66/M	Myocardial revascularization	Left pectoralis major muscle flap	7	Seroma	7
67/M	Valve prosthesis placement	Left pectoralis major muscle flap	14	Seroma, granuloma	19
21/M	Dental infection/Ludwig's syndrome	Left pectoralis major muscle flap	8	Seroma, pain, partial dehiscence	9

M=male; F=female

Harvesting a flap from the rectus abdominis fascia was necessary in two patients, in association with the pectoralis major muscle flap, to facilitate the closure of the distal wound. As shown in Table 1, all flaps were dissected from the left pectoralis major muscle.

In the postoperative period, seroma discharge from the surgical wound was observed in six patients, five reported intense pain (temporary), three had partial cutaneous dehiscence, and two presented granuloma of the incision. After 3 months of reconstruction, all patients presented stiffness at the sternal region observed during palpation, and after 6 months no patient had chest instability or complain of reduction of muscle strength from the left arm (flap side). Computed tomography images show the sternal cleft filled by the muscle flap in the later postoperative period (Figures 2D and 3D).

A new surgical approach was necessary in only one case due to subcutaneous granuloma in the distal third of the incision, in which a small rhomboid skin flap was performed, presenting a proper evolution, without the need for a new muscle flap (Figure 3). There were no deaths related to the sternal wound dehiscence reconstruction procedure.

## DISCUSSION

Sternal wound dehiscence has been classified as a complex wound, and its treatment is a challenge for surgeons^[12]^. Several surgical options have been proposed for the treatment of sternal wound dehiscence, including primary synthesis with metal wires, titanium plates and sternal reconstruction using muscle or omental flaps^[3,4,6,13,14]^. The bilateral pectoralis major muscle flaps are related to better rates of stabilization of the chest wall when compared to sternal reconstruction with metal wires^[3]^. Eventually, a portion of the anterior fascia of the rectus abdominis muscle can be dissected and attached to the distal third of the pectoralis major muscle flap to facilitate the wound closure, or also by transposing solely the cephalic portion of the rectus abdominis muscle, leading to a decreased risk of postoperative herniation of the abdominal wall^[9]^. Kaláb et al.^[5]^ used allogenic bone graft associated with the pectoralis major flap to improve the stabilization of the thoracic wall in patients of larger sternal defects.

Latissimus dorsi or rectus abdominis flaps may also be used. Such muscles have reliable vascularization and can cover major defects, or they can be used associated with the pectoralis major muscle flap. However, application of these flaps demands longer surgical time, more laborious dissection and extra incisions. Besides, some complications may take place, for example the deficit of flap perfusion (leading to flap necrosis), the weakening of the abdominal wall, etc.^[2,6]^.

Another option for reconstruction of these defects is the fasciocutaneous flap based on perforator vessels from the internal thoracic artery, as described by Koulaxouzidis et al.^[15]^. It is a less aggressive therapeutic option, associated with good aesthetic results and preservation of the breath muscles.

The omental flap can also be used for the reconstruction of sternal defects; however its surgical technique is complex and it is associated with an increased mortality rate. Besides, the omental flap does not lead to sternal stabilization and it does not have skin coverage. Its main indication is related to situations in which other flaps have failed^[2,6,16]^.

The reconstruction with the pectoralis major muscle flap mobilized bilaterally has been the first choice in several centers, since it does not show greater adverse effects on pulmonary function and even improves the parameters of spirometry, cosmetic results and thoracic stabilization^[2,3,6,7]^. However, it may lead to impairment to execute hobbies and social activities, as pointed by Klesius et al.^[6]^, after analyzing the results from a quality of life questionnaire.

Despite the several surgical treatment options previously described, the use of the pectoralis major muscle flap mobilized unilaterally (although less frequent) can be an interesting option, associated or not to other muscle or omental flaps^[3,9]^.

The unilateral pectoralis major muscle flap is a more conservative technique when compared to the bilateral pectoralis muscle flap, in order to minimize possible functional alterations due to the donor area deficit. Besides, it presents shorter surgical time, and a low rate of complications as evidenced in the cases reported in the present article. In a study performed by Fernández-Palacios et al.^[11]^, in 2010, the results of unilateral and bilateral pectoralis major muscle flaps for the treatment of mediastinitis were compared. They found that unilateral flap had similar results to the bilateral flap related to morbidity, mortality and complications. However, the unilateral technique was faster (shorter surgical time, *P*=0.001), there was less need of postoperative blood transfusions and the extubation was earlier. The authors believe that the unilateral flap is a low aggressive surgery, with the preservation of the contralateral pectoralis muscle^[11]^.

The unilateral pectoralis major muscle flap can also be used in children without functional deficit, with good cosmetic results^[17]^. A possible disadvantage of this approach, an opinion shared with Spartalis et al.^[18]^, would be the limited arc of rotation of the pectoralis major muscle flap to adequately cover the lower third of the sternum defect.

As we noted in our experience, the use of unilateral pectoralis major muscle flap showed partial skin dehiscence rate of 27.3%, and length of stay of about three weeks, similar to that found by Carlesimo et al.^[13]^ (18 days of hospitalization after reconstruction with bilateral pectoralis major muscle flap).

We observed that rotation of just one pectoralis major muscle flap was sufficient for the sternal cleft closure in all the eleven patients, and did not require a second pectoralis muscle flap. The greatest difficulty of closure is located at the distal wound. However, a second pectoralis major muscle flap would not satisfy this purpose, given the equally limited arc of rotation. In two patients, we chose to associate small flaps of the rectus abdominis fascia to better anchor the suture of the distal and medial border of the pectoralis major muscle flap, in an attempt to complete obliteration of the sternal cleft.

As mediastinal wound infection after sternotomy has been associated with high mortality rates^[19-22]^, its treatment requires an early aggressive debridement and reconstruction. For this, muscle flap coverage has become the gold standard procedure^[9,11]^. The present study evidenced that the unilateral pectoralis major muscle flap is a satisfactory option for wound closure resulting from sternotomy dehiscence, with low morbidity and acceptable aesthetic and functional results, providing stability of the sternal region. The stiffness noted on palpation of the sternal region after a few months postoperatively is probably due to the fibrosis that is established in the sternal cleft, now occupied by the volume from the muscle flap.

## CONCLUSION

The complex wound from sternotomy dehiscences presents itself as a challenge to surgical teams. Treatment should include debridement of necrotic tissue and preferably coverage with well-vascularized tissue. We propose that the unilateral pectoralis major muscle flap is an interesting and low morbidity option for the reconstruction of sternal wound dehiscences, with proper sternum stability and satisfactory functional and aesthetic outcomes.

**Table:** 

Authors' roles & responsibilities	
GSH	Substantial contributions to the conception or design of the work; or the acquisition, analysis, or interpretation of data for the work; final approval of the version to be published
PSC	Substantial contributions to the conception or design of the work; or the acquisition, analysis, or interpretation of data for the work; final approval of the version to be published
AAN	Substantial contributions to the conception or design of the work; or the acquisition, analysis, or interpretation of data for the work; final approval of the version to be published
JBA	Substantial contributions to the conception or design of the work; or the acquisition, analysis, or interpretation of data for the work; final approval of the version to be published
VZS	Substantial contributions to the conception or design of the work; or the acquisition, analysis, or interpretation of data for the work; final approval of the version to be published
IRA	Substantial contributions to the conception or design of the work; or the acquisition, analysis, or interpretation of data for the work; final approval of the version to be published
AJR	Substantial contributions to the conception or design of the work; or the acquisition, analysis, or interpretation of data for the work; final approval of the version to be published
JAFJ	Substantial contributions to the conception or design of the work; or the acquisition, analysis, or interpretation of data for the work; final approval of the version to be published
